# Can the Oxygen Saturation Index Predict Severe Bronchopulmonary Dysplasia?

**DOI:** 10.3390/children12050582

**Published:** 2025-04-30

**Authors:** Hulya Ozdemir, Sinem Gulcan Kersin, Asli Memisoglu, Ibrahim Kandemir, Hulya Selva Bilgen

**Affiliations:** 1 Division of Neonatology, Department of Pediatrics, Marmara University Pendik Training and Research Hospital, Istanbul 34899, Turkey; sinemgulcankersin@gmail.com (S.G.K.); acinarmemisoglu@gmail.com (A.M.); hulya.bilgen@gmail.com (H.S.B.); 2 Department of Pediatrics, Faculty of Medicine, Biruni University, Istanbul 34295, Turkey; ibrahimkandemir@gmail.com

**Keywords:** preterm infant, severe bronchopulmonary dysplasia, death, oxygen saturation index

## Abstract

**Background/Objectives**: Even with improvements in perinatal care, bronchopulmonary dysplasia (BPD) continues to be a major challenge, especially in smaller and more premature infants. Early detection of severe BPD can improve treatment outcomes. This study aims to evaluate the correlation between the oxygen saturation index (OSI) and severe BPD/death in preterm infants, with a focus on the OSI’s predictive value. **Methods**: In this retrospective observational study, infants with a gestational age of less than 32 weeks who required either invasive or non-invasive mechanical ventilation were included. Ventilator settings and OSI values were collected on days 3, 7, 14, 21, and 28 of life. The correlations between postnatal OSIs and outcomes such as death or severe BPD were analyzed using logistic regression. **Results**: Out of the 210 eligible infants, 54 (25.7%) either died or were diagnosed with severe BPD. In our study, OSI values on postnatal days 14, 21, and 28 were significantly higher in preterm infants who developed severe BPD or died, with mean OSI-14, OSI-21, and OSI-28 values of 4.9, 3.5, and 2.8, respectively. The OSI showed the highest sensitivity and specificity on postnatal days 14 and 21, with cut-off points of 3.6 and 3.1, respectively. We built a basic chart to predict severe BPD/death with OSI-14 and OSI-21 and delivery room intubation with 86% sensitivity and 84.5% specificity (increasing up to 98.8% specificity). **Conclusions**: This study showed that the diagnostic power of the OSI in predicting severe BPD or death was highest for OSI-14 and OSI-21. We demonstrated that calculating the OSI, a non-invasive clinical tool, can predict severe BPD/death in infants born before 32 weeks as early as the 14th day of life.

## 1. Introduction

Despite significant advancements in perinatal care, bronchopulmonary dysplasia (BPD) remains one of the most common, complex, and challenging conditions in perinatal medicine. The underlying mechanisms of BPD are still not completely understood [1]. The population of infants at risk for BPD has changed over the past few decades; smaller, more immature infants are now at the highest risk [2]. In this context, infants at the greatest risk for developing BPD, particularly those born before 30 weeks of gestation, are often exposed to supplemental oxygen during the late canalicular or saccular stages of lung development. The impact of genetic factors, both intrauterine and postnatal inflammation and infection, oxidative stress, and neonatal intensive care unit care practices on disrupting lung development continues to be investigated [3].

Considering that infants with severe BPD often require extended mechanical ventilation and face long-term challenges related to pulmonary and neurodevelopmental outcomes, research focused on identifying predictive factors for the early diagnosis of severe BPD is crucial [4]. Early and accurate detection of severe BPD can enable the timely initiation of treatments when they are most likely to be effective. Unfortunately, there are limited evidence-based therapies available for the prevention and treatment of BPD [5,6,7].

Any change in the severity of lung disease is reflected as a change in the need for distending pressure, the fraction of inspired oxygen (FiO_2_), or both. A tool incorporating these parameters could potentially aid in the objective assessment of lung disease severity [8].

The oxygen saturation index (OSI), a non-invasive clinical tool, has been utilized to monitor the severity of lung disease in neonates undergoing both non-invasive and invasive mechanical ventilation [9,10,11,12,13,14]. Therefore, the OSI is highlighted as an objective and non-invasive tool that provides rapid and continuous bedside assessment, making it one of the promising methods.

Besides being a valuable bedside tool, the OSI ensures consistency in expressing the severity of illness, which is advantageous for research purposes. It has been confirmed that the OSI correlates with the oxygenation index (OI) and the alveolar–arterial oxygen gradient, thus minimizing the need for invasive blood gas measurements [11,15,16]. In neonates with hypoxemic respiratory failure, the oxygen saturation index is proposed as a reliable alternative that uses oxygen saturation (SpO_2_) instead of PaO_2_ [9,10,11,17]. As such, the non-invasive clinical tool OSI could continuously monitor the infant’s respiratory status and predict adverse neonatal outcomes [9,10,11,17,18,19,20].

Based on a review of the current literature, it is evident that the search for non-invasive clinical respiratory tools to predict bronchopulmonary dysplasia is ongoing. To date, reports on the clinical use of the OSI in critically ill neonates are scarce, and there are no reports available specifically for bronchopulmonary dysplasia. This study primarily aims to assess the correlation between the oxygen saturation index and severe BPD/death in preterm infants, demonstrate the predictive value of the OSI for severe BPD/death, and secondarily aims to identify clinical features associated with severe BPD/death.

## 2. Materials and Methods

This was a retrospective observational study conducted in the neonatal intensive care unit at Marmara University Hospital between January 2018 and December 2023. Approval for this study was obtained from the Ethics Committee of Marmara University School of Medicine (Number 28.06.24.730).

The study population consisted of preterm infants with a gestational age of less than 32 weeks who required either invasive or non-invasive mechanical ventilation. Infants who died due to only respiratory causes between the 8th day of life and 36 weeks of PMA or until discharge were also included in the study. The exclusion criteria for the study consisted of infants with major congenital malformations, those who died within the first seven days of life, between the 8th day of life and postmenstrual 36 weeks/discharge from non-respiratory causes, and those who did not receive any form of mechanical ventilation [21].

All infants who required surfactant instillation were intubated, or minimally invasive surfactant therapy techniques were used throughout the study period. During the study, the 2020 American Heart Association guidelines for cardiopulmonary resuscitation and emergency cardiovascular care were applied in newborn resuscitation [22]. Early nasal CPAP was applied to preterm infants who did not require intubation in the delivery room. These infants were provided with non-invasive respiratory support in the neonatal intensive care unit unless they required intubation based on their respiratory status and blood gases.

In preterm infants, to prevent over-distension, atelectasis, and subsequent injury, we typically initiate mechanical ventilation with rates of 40–60 breaths per minute, an inspiration time of 0.25–0.35 s, and a tidal volume of 4–6 mL/kg. For high-frequency oscillation ventilation, we use the minimum mean airway pressure (MAP) and amplitude necessary to meet clinical objectives. In our study, oxygen saturation targets for preterm infants under 32 weeks were implemented at 90–94% according to the European Consensus Guidelines on the Management of Respiratory Distress Syndrome [23]. Based on this information, the oxygen saturation alarm limits for premature babies were set to 89–95%.

Extubation was considered in patients who showed the following minimal ventilator settings for >12 h according to the protocol of our center: MAP ≤ 8 cm H_2_O; FiO_2_ ≤ 0.4; and mandatory respiratory frequency ≤ 25/min. In this study, non-invasive mechanical ventilation (nCPAP/nIPPV) was applied to preterm infants post-extubation.

The charts of the study population were reviewed. Demographic, perinatal, and neonatal data including gestational age, birth weight, sex, small for gestational age (SGA), delivery room intubation, postnatal systemic steroid use, mechanical ventilation duration, length of hospital stay, necrotizing enterocolitis (NEC) stage ≥ 2B, HsPDA (hemodynamically significant patent ductus arteriosus), IVH (intraventricular haemorrhage) grade ≥ 2, and retinopathy of prematurity (ROP) (requiring treatment) were recorded.

The ventilator settings on days 3, 7, 14, 21, and 28 of life were collected, and the OSI was calculated on the same days. The oxygenation saturation index (OSI) was calculated as follows: OSI = FiO_2_ [%] × MAP [cm H_2_O]/SpO_2_ [%] [10]. The mean airway pressure was calculated as MAP = PEEP + ((PIP − PEEP) × (t_i_/t_i_ + t_e_)), where PIP is the peak inspiratory pressure, t_i_ is the inspiratory time, and t_e_ is the expiratory time [24]. The daily median values of PIP, PEEP, FiO_2_, and SpO_2_ were used in the OSI calculation. For continuous positive airway pressure, PEEP was considered equivalent to MAP [25].

Based on the current most commonly accepted definition of BPD as the need for oxygen supplementation at 36 weeks corrected gestational age, the National Institute of Child Health criteria were used to define BPD and classify it as mild, moderate, or severe based on FiO_2_ or positive pressure ventilation requirements. Mild BPD was defined as the ability to breathe room air, moderate BPD as needing an FiO_2_ of less than 0.30, and severe BPD as needing an FiO_2_ of 0.30 or higher, or positive pressure ventilation, at a postmenstrual age of 36 weeks [26].

### 2.1. Sample Size

Although this study is a retrospective observational study, the sample size was also calculated based on an estimated severe BPD rate of approximately 30% and a minimum OSI value of 3.1, which indicates hypoxic respiratory failure according to previous studies [27,28]. With a Type I error (alpha) of 0.05, a power (1-beta error) of 0.9, and an effect size (d) of 0.5, we calculated that a minimum total of 52 in the severe BPD cases was required. G*Power 3.1 software was used for this calculation. Nevertheless, given the retrospective design of our study, we included a greater number of cases than originally calculated. We included all patients that we could reach in the hospital records between the defined study dates.

### 2.2. Statistical Analysis

The statistical analysis of the data was performed using Stata statistical software (StataCorp, 2021. Release 17, College Station, TX, USA). The demographic and clinical characteristics of the patients were presented as number (N), percentage (%), mean ± standard deviation, median, and range values. The normality of the distribution of continuous variables was assessed using the Kolmogorov–Smirnov and Shapiro–Wilk tests. Patients were grouped according to the development of BPD or mortality, and the relationships between individual characteristics and patient groups were examined using the Mann–Whitney U test, Chi-square test, and Fisher’s exact test. We also built multivariate analysis using a generalized linear model using clinical and OSI measurement results as possible confounders. We picked the most significant factor (confounding factor) using *backward elimination and forward selection methods*. The predictive value of the OSI for severe BPD/death was analyzed using Receiver Operating Characteristic (ROC) curves. The Area Under the Curve (AUC) was calculated for days 3, 7, 14, 21, and 28. The optimal threshold for each day was determined based on the highest Youden index. Statistical significance was set at *p* < 0.05 for all analyses.

## 3. Results

**Figure 1:**
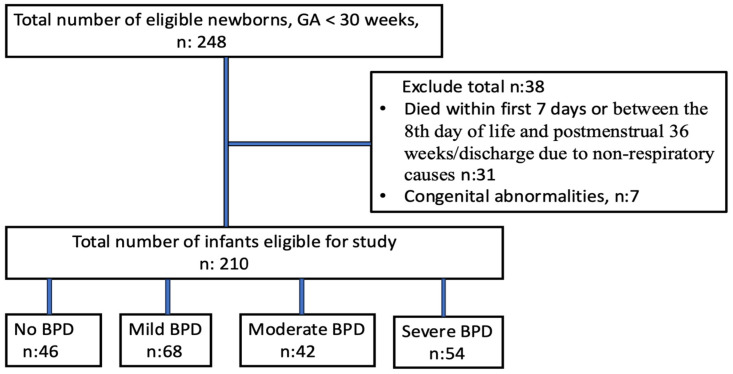
Flow diagram of study population.

In our study, 248 infants were born at less than 32 weeks of gestation. Among them, 31 died within the first seven days of life or between the eighth day of life and postmenstrual 36 weeks/discharge due to non-respiratory causes, seven infants had major congenital abnormalities, leaving 210 infants for evaluation. Eleven infants died between the 8th day of life and postmenstrual 36 weeks/discharge and they died only due to respiratory causes. The flow diagram of the study population is shown in Figure 1.

The demographic features of these 210 infants are shown in Table 1. In our study, the rate of severe BPD/death was found to be 25.7%. The basal demographic features and clinical data of infants with and without severe BPD/death are compared in Table 2. As expected, infants with severe BPD were born at an early gestational age and had a smaller birth weight (*p* < 0.001). In addition, chorioamnionitis, delivery room intubation, IVH grade ≥ 2, NEC stage ≥ 2B, ROP (requiring treatment), HsPDA, late postnatal steroid use, invasive or non-invasive respiratory support duration, and hospital stay were found to be statistically significantly higher in infants with severe BPD (Table 2). We subjected all factors (except the OSI results) to a multivariate analysis to identify the most significant predictors of severe BPD or death. Delivery room intubation was associated with a 2.6-fold increased risk (95% CI: 0.9–7.4), and hemodynamically significant PDA (HsPDA) was associated with a 2.5-fold increased risk (95% CI: 0.9–6.7) of severe BPD or death. Each additional day of invasive mechanical ventilation was associated with a 1.16-fold (95% CI: 1.09–1.26) increase in risk, and each additional day of non-invasive mechanical ventilation with a 1.04-fold (95% CI: 1.02–1.06) increase in risk (generalized linear model, R^2^: 0.585).

The comparison of OSI-3, OSI-7, OSI-14, OSI-21, and OSI-28 between the two groups is shown in Table 3. The mean OSI-14, OSI-21, and OSI-28 values were found to be 4.9, 3.5, and 2.8, respectively, in this study. These values were significantly higher in preterm infants with severe BPD compared with those without BPD or with mild/moderate BPD on postnatal days 14, 21, and 28 (Table 3).

As we subjected the five total OSI results (transformed values) to another multivariate model, the 14th and 21st days remained as significant confounders for predicting severe BPD/death. Every 1 point increase in OSI-14 increased severe BPD/death by 1.5 (0.99–2.48) times, and every 1 point increase in OSI-21 by 3.88 (1.95–8.46) times.

**Figure 2:**
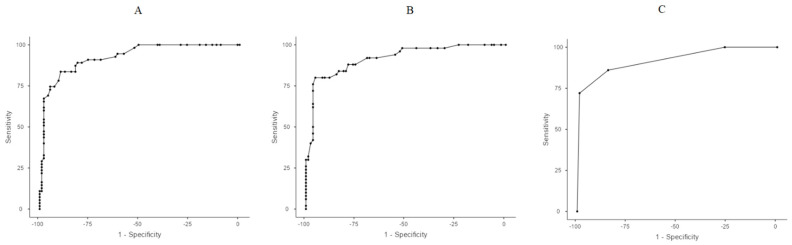
Receiver Operating Characteristic (ROC) curves. (**A**) ROC analysis of OSI values on OSI-14. (**B**) ROC analysis of OSI values on OSI-21. (**C**) ROC analysis for OSI-14 > 3.6, OSI-21 > 3.0, and delivery room intubation.

When OSI values were combined with the aforementioned predictors in an additional multivariate model, OSI-14 and OSI-21, along with intubation in the delivery room, remained as the most significant predictive factors. A Receiver Operating Characteristic (ROC) analysis was conducted using these three variables to determine optimal cut-off points, prioritizing sensitivity. The selected threshold for OSI-14 to reach the best sensitivity was 3.6, while for OSI-21 it was 3.0 (Table 4). Using these cut-off values, a scoring system was developed, assigning 1 point for each of the following criteria: OSI-14 > 3.6, OSI-21 > 3.0, delivery room intubation. The total score (ranging from 0 to 3) demonstrated 86% sensitivity and 84.5% specificity for a threshold of ≥2 points, whereas a threshold of ≥3 points yielded 72% sensitivity and 98.8% specificity. These findings indicate that OSI-14 and OSI-21, along with intubation in the delivery room, are strong predictors of severe BPD/death (Table 4, Figure 2).

## 4. Discussion

In our study, we evaluated the relationship between severe BPD/death and the OSI within the first 28 days in premature infants born before 32 weeks of gestation. In particular, we found a significant relationship between the OSI and severe BPD/death on the 14th and 21st days of life. Advances in neonatal care have not reduced the incidence of BPD and no new breakthrough therapy has been successfully translated into the clinic in recent decades [29]. In recent years, interventions such as stem cell therapy have been proposed as promising treatments to prevent BPD in extremely preterm infants [30]. To identify evolving BPD early and administer preventive treatments earlier, several predictive clinical tools have been developed [31,32,33,34,35,36]. However, we believe that there is still a need for respiratory clinical tools to achieve earlier diagnosis. In this study, we aimed to emphasize that the OSI, which is used in the diagnosis of respiratory failure in pediatrics, may also be effective in identifying severe BPD/death. This study suggests that promising preventive treatments for BPD could be administered to infants as early as postnatal day 14.

The oxygen saturation index involves measuring the respiratory support provided to the newborn through FiO_2_ and MAP, as well as the response to this support measured by SpO_2_ [13]. The OSI has been used to indicate the severity of respiratory disease in newborns receiving both invasive and non-invasive respiratory support. It has been shown to reduce the need for invasive blood gas sampling and has been validated through studies comparing it with the alveolar–arterial oxygen gradient and OI for clinical use [9,13,14,15,16].

Thandaveshwara et al. [13] demonstrated that in newborns who required invasive mechanical ventilation due to progressive respiratory disease while being monitored with non-invasive mechanical ventilation, the mean OSI values were found to be 1.88 and the OSI was shown that have a significant positive correlation of 82.7% against A-aDO2 and can be a valuable tool to assess respiratory distress in neonates without arterial blood gas. In another study, the correlation between the OI and OSI was evaluated in newborns receiving respiratory support, and it was reported that an OSI value of <2 indicates mild respiratory disease, 2–3.7 indicates moderate, and >3.7 indicates severe respiratory disease [14].

Sunil et al. [26] also identified that the OSI was also used to assess the severity of hypoxic respiratory failure in premature infants receiving invasive respiratory support. It was shown that an OSI value of ≤3 indicates mild, 3.1–6.5 indicates moderate, and >6 indicates severe respiratory failure.

The mean OSI values in premature infants with severe BPD/death were found to be 2.7, 2.6, 4.9, 3.5, and 2.8 on postnatal days 3, 7, 14, 21, and 28, respectively. It was observed that the OSI on postnatal days 14, 21, and 28 was significantly higher in the severe BPD/death group compared with those without severe BPD/death. In our study, the cut-off values for OSI-14 and OSI-21 in the severe BPD/death group were found to be 3.6 and 3.0, respectively. These values demonstrated significantly higher sensitivity and specificity compared with other days. We would like to emphasize again that these are the most specific and sensitive days for predicting severe BPD/death. We determined that the OSI-14 and 21 values predicting severe BPD/death were at least 3.6/3.0 in this study. We emphasize that 3.6/3.0 were not the best thresholds on their own, but they predicted better in the new-built chart.

Chou et al. [25] evaluated the OI values of infants during the first 3 weeks of life to predict moderate-to-severe BPD and found an average OI value of 5 in the severe BPD group. According to OI and OSI validation studies, an OI of 5 corresponds to an OSI of 3.0, an OI of 10 corresponds to an OSI of 5.3, and an OI of 15 corresponds to an OSI of 7.7 [16]. Based on this information, it is understood that, in Chou and colleagues’ study, the OSI value predicting moderate-to-severe BPD is approximately 3 [25]. In our study, the OSI values in infants of severe BPD/death were found to be highest between 4.9 and 3.5, whereas according to the findings of Chou et al., the OI values were determined to be between 5 and 10.

In 2019, Jung et al. [37] evaluated the predictive value of the respiratory severity score calculation tool for severe BPD/death and respiratory severity score is obtained as a percentage of the product of MAP and FiO_2_ in invasively ventilated infants in this study. Jung et al. [37] found values of ≥3 on postnatal day 14, ≥3.6 on day 21, and ≥3.2 on day 28. In our study, values were significantly higher on days 14, 21, and 28, with a minimum threshold of ≥2.8. A distinct aspect of our study is that OSI values were also obtained for non-invasively ventilated infants. Furthermore, our study demonstrated that changes in the OSI were greater in the severe BPD/death group OSI-14, OSI-21, and OSI-28. In our study, the reason for finding the highest OSI values on postnatal days 14, 21, and 28 may be related to intermittent hypoxemia observed in preterm infants. The increase in intermittent hypoxemia during the 2nd to 6th weeks is associated with the developmental maturation of the respiratory system and lung injury, making intermittent hypoxemia more prominent [38]. It has been shown in another study that prolonged intermittent hypoxemia beginning in the first week after birth was associated with an increased risk of developing severe BPD [39]. Although oxygen saturation histograms could not be presented in our study, we considered that this could be one of the reasons for the higher OSI-14 compared with other days. As we well know, BPD develops through multiple factors, with lung injury resulting from an imbalance between pro-inflammatory and anti-inflammatory pathways. This imbalance can occur in various conditions, including prenatal factors such as chorioamnionitis and postnatal influences such as mechanical ventilation, relative hyperoxia, and sepsis. Systemic corticosteroids possess anti-inflammatory effects and offer clinicians a means to prevent, manage, or reduce lung damage [21]. In our study, we showed that late postnatal steroids were more common in the severe BPD/death group. However, we did not find a correlation between OSI-14, OSI-21, and OSI-28 and postnatal steroids.

In this study, the NICHD 2001 definition of BPD was used [26]. This definition applies to infants born before 32 weeks of gestation who require oxygen support. Infants diagnosed with severe BPD are those who require non-invasive or invasive mechanical ventilation or more than 30% oxygen support at 36 weeks postmenstrual age [26]. Based on this definition, we believe that the OSI, which incorporates oxygen saturation in its calculation, may demonstrate superiority over the respiratory severity score in predicting infants at risk of severe BPD. In 2021, the NICHD developed an updated prediction tool for BPD/death. In this new BPD prediction calculator, the most important risk factor for predicting BPD/death on postnatal day 1 was found to be birth weight, while respiratory support was the key determinant on days 3, 7, 14, and 28. Other significant risk factors identified on days 14 and 28 included FiO_2_ and undergoing NEC surgery [36].

In our study, the most significant risk factors for severe BPD/death included gestational age, birth weight, SGA, delivery room intubation, IVH grade ≥ 2, NEC stage ≥ 2B, ROP (requiring treatment), HsPDA, and the duration of both invasive and non-invasive mechanical ventilation.

When multivariate analysis was applied to these risk factors, delivery room intubation, HsPDA, and the duration of both invasive and non-invasive mechanical ventilation were found to be significantly correlated with severe BPD/death.

Other studies on this subject have also identified similar risk factors. Among all important perinatal and postnatal factors, the delivery room intubation was found to be the most predictive clinical factor on days 14 and 21, based on OSI measurements. Therefore, early CPAP application in the delivery room and timely extubation are well-known practices that help reduce lung injury in premature infants [23].

A major limitation of our study is its retrospective design. Another limitation of our study is the absence of oxygen saturation histograms. In particular, the lack of these data prevents a clear demonstration of the relationship between low oxygen saturation, intermittent hypoxemia, and severe BPD/death. Additionally, the single-center nature of our study is another significant limitation. We believe that larger, multicenter, and prospective studies would be more valuable in evaluating the relationship between the OSI and severe BPD/death.

## 5. Conclusions

Our study showed that the oxygen saturation index on postnatal days 14 and 21 predicted severe BPD/death in premature infants born before 32 weeks of gestation. The sensitivity and specificity of the OSI in predicting severe BPD/death were highest on postnatal days 14 and 21. In conclusion, we believe that calculating the OSI, a non-invasive clinical tool, can predict severe BPD/death in infants born before 32 weeks as early as the 14th day of life. This early prediction may allow for timely treatment decisions for high-risk preterm babies, potentially including promising therapies such as stem cell treatment.

## Figures and Tables

**Table 1:** Clinical and demographic characteristics of the study patients.

	N = 210
Gestational age (days)	28.1 ± 2.9
Birth weight (g), mean ± SD	1105 ± 405
Gender (F), n (%)	107 (51.2)
Delivery mode (VD), n (%)	68 (32.4)
SGA, n (%)	23 (11.0)
Antenatal steroid, n (%)	113 (53.8)
Chorioamnionitis, n (%)	10 (4.8)
Delivery room intubation, n (%)	122 (58.1)
IVH grade ≥2, n (%)	26 (12.4)
NEC stage ≥2B, n (%)	21 (10.0)
ROP (requiring treatment), n (%)	21 (10.0)
HsPDA n (%)	93 (44.3)
Late postnatal steroid, n (%)	51 (24.3)
İnvasive mechanical ventilation duration (days)	4 (0–21)
Non-invasive mechanical ventilation duration (days)	28 (6–54)
BPD, n (%)	164 (78.1)
Mild BPD, n (%)	68 (32.3)
Moderate BPD, n (%)	42 (20.0)
Severe BPD, n (%)	54 (25.7)
Hospital stay (days), mean ± SD	63 (37–103)

Data are presented as median (interquartile range), mean ± standard deviation, and n (%). VD: vaginal delivery, SGA: small for gestational age, IVH: intraventricular hemorrhage, NEC: necrotizing enterocolitis, ROP: retinopathy of prematurity, PDA: patent ductus arteriosus, BPD: bronchopulmonary dysplasia.

**Table 2:** Clinical and demographic features of patients with severe BPD/death vs. without severe BPD.

	Infants Without Severe BPD (n = 156)	Infants with Severe BPD/Death (n = 54)	*p*
Gestational age (days)	29.0 ± 2.0	25.9 ± 3.4	<0.001
Birth weight (g), mean ± SD	1273 ± 373	777 ± 232	<0.001
Gender (F), n (%)	74 (49.6)	33 (54.1)	0.759
Delivery mode (VD), n (%)	47 (31.5)	20 (32.8)	0.160
SGA, n (%)	12 (8.1)	11 (18.0)	0.135
Antenatal steroid, n (%)	78 (52.3)	34 (55.7)	0.449
Chorioamnionitis, n (%)	4 (2.7)	6 (9.8)	0.022
Delivery room intubation, n (%)	67 (44.9)	54 (88.5)	<0.001
IVH grade ≥2, n (%)	11 (7.4)	15 (24.6)	<0.001
NEC stage ≥2B, n (%)	5 (3.4)	15 (24.6)	<0.001
ROP (requiring treatment), n (%)	6 (4.0)	15 (24.6)	<0.001
HsPDA n (%)	45 (30.2)	47 (77.0)	<0.001
Late postnatal steroid, n (%)	15 (10.1)	35 (57.4)	<0.001
İnvasive mechanical ventilation duration (days), median (range min–max)	4 (0– 101)	9 (3–260)	<0.001
Non-invasive mechanical ventilation duration (days), mean ± SD/median (range min–max)	18 (3–115)	31 (0–153)	<0.001
Hospital stay (days), mean ± SD	62.4 ± 36.7	112.5 ± 65.0	<0.001

VD: vaginal delivery, SGA: small for gestational age, IVH: intraventricular hemorrhage, NEC: necrotizing enterocolitis, ROP: retinopathy of prematurity, PDA: patent ductus arteriosus, BPD: bronchopulmonary dysplasia.

**Table 3:** OSI values on postnatal days 3, 7, 14, 21, and 28 in infants with severe BPD versus without severe BPD.

OSI (Postnatal Days)	Infants Without Severe BPD	Infants with Severe BPD	*p*
3	2.1 (1.3–2.4)	2.7 (2.2–3.7)	0.07
7	2.0 (1.1–2.2)	2.6 (2.1–3.4)	0.06
14	1.3 (0.9–2.2)	4.9 (3.5–6.6)	<0.001
21	1.3 (0.9–1.9)	3.5 (3.1–4.4)	<0.001
28	1.4 (0.8–1.9)	2.8 (2.2–3.7)	<0.001

**Table 4:** Significant ROC results for OSI-14, OSI-21, delivery room intubation, and the scoring chart.

		Sensitivity	Specificity	PPV	NPV	AUC	Metric Score
OSI-14	3.6	89.09%	81.05%	73.13%	92.77%	0.93	1.7
	3.4	87.27%	82.11%	73.85%	91.76%	0.93	1.69
	3.3	83.64%	87.37%	79.31%	90.22%	0.93	1.71
	3.2	83.64%	89.47%	82.14%	90.43%	0.93	1.73
	2.6	78.18%	90.53%	82.69%	87.76%	0.93	1.69
	2.5	74.55%	94.74%	89.13%	86.54%	0.93	1.69
OSI-21	2.8	80%	90.59%	83.33%	88.51%	0.92	1.71
	2.9	80%	91.76%	85.11%	88.64%	0.92	1.72
	3	80%	95.29%	90.91%	89.01%	0.92	1.75
	3.1	76%	96.47%	92.68%	87.23%	0.92	1.72
Delivery room intubation	2	94.55%	54.84%	42.62%	96.59%	0.75	1.49
OSI-14 < 3.6 + OSI-21 < 3.0 + Delivery room intubation	2	86%	84.52%	76.79%	91.03%	0.92	1.71
	3	72%	98.81%	97.30%	85.57%	0.92	1.71

## Data Availability

The original contributions presented in the study are included in the article, further inquiries can be directed to the corresponding author.

## Abbreviations

The following abbreviations are used in this manuscript:

BPD	Bronchopulmonary dysplasia
OSI	Oxygen saturation index
FiO_2_	Fraction of inspired oxygen
SpO_2_	Oxygen saturation
MAP	Mean airway pressure
F	Gender
SGA	Small for gestational age
NEC	Necrotizing enterocolitis
HsPDA	Hemodynamically significant patent ductus arteriosus
IVH	Intraventricular hemorrhage
ROP	Retinopathy of prematurity

## References

[1] Stoll B.J., Hansen N.I., Bell E.F., Walsh M.C., Carlo W.A., Shankaran S., Laptook A.R., Sánchez P.J., Van Meurs K.P., Wyckoff M. (2015). Eunice Kennedy Shriver National Institute of Child Health and Human Development Neonatal Research Network. Trends in Care Practices, Morbidity, and Mortality of Extremely Preterm Neonates, 1993–2012. JAMA.

[2] O’Reilly M., Sozo F., Harding R. (2013). Impact of preterm birth and bronchopulmonary dysplasia on the developing lung: Long-term consequences for respiratory health. Clin. Exp. Pharmacol. Physiol..

[3] Higgins R.D., Jobe A.H., Koso-Thomas M., Bancalari E., Viscardi R.M., Hartert T.V., Ryan R.M., Kallapur S.G., Steinhorn R.H., Konduri G.G. (2018). Bronchopulmonary Dysplasia: Executive Summary of a Workshop. J. Pediatr..

[4] Lal C.V., Ambalavanan N. (2015). Biomarkers, Early Diagnosis, and Clinical Predictors of Bronchopulmonary Dysplasia. Clin. Perinatol..

[5] van de Loo M., van Kaam A., Offringa M., Doyle L.W., Cooper C., Onland W. (2024). Corticosteroids for the prevention and treatment of bronchopulmonary dysplasia: An overview of systematic reviews. Cochrane Database Syst. Rev..

[6] Poets C.F., Lorenz L. (2018). Prevention of bronchopulmonary dysplasia in extremely low gestational age neonates: Current evidence. Arch. Dis. Child. Fetal Neonatal Ed..

[7] Horbar J.D., Edwards E.M., Greenberg L.T., Morrow K.A., Soll R.F., Buus-Frank M.E., Buzas J.S. (2017). Variation in Performance of Neonatal Intensive Care Units in the United States. JAMA Pediatr..

[8] Tan Y.W., Adamson L., Forster C., Davies B., Sharkey D. (2012). Using serial oxygenation index as an objective predictor of survival for antenatally diagnosed congenital diaphragmatic hernia. J. Pediatr. Surg..

[9] Muniraman H.K., Song A.Y., Ramanathan R., Fletcher K.L., Kibe R., Ding L., Biniwale M. (2019). Evaluation of oxygen saturation index compared with oxygenation index in neonates with hypoxemic respiratory failure. JAMA Netw. Open.

[10] Khalesi N., Choobdar F.A., Khorasani M., Sarvi F., Haghighi Aski B., Khodadost M. (2019). Accuracy of oxygen saturation index in determining the severity of respiratory failure among preterm infants with respiratory distress syndrome. J. Matern. Fetal Neonatal Med..

[11] Rawat M., Chandrasekharan P.K., Williams A., Gugino S., Koenigsknecht C., Swartz D., Ma C.X., Mathew B., Nair J., Lakshminrusimha S. (2015). Oxygen saturation index and severity of hypoxic respiratory failure. Neonatology.

[12] Thomas N.J., Shaffer M.L., Willson D.F., Shih M.C., Curley M.A. (2010). Defining acute lung disease in children with the oxygenation saturation index. Pediatr. Crit. Care Med..

[13] Thandaveshwara D., Chandrashekar Reddy A.H., Gopalakrishna M.V., Doreswamy S.M. (2021). Saturation oxygenation pressure index: A non-invasive bedside measure for severity of respiratory disease in neonates on CPAP. Eur. J. Pediatr..

[14] Doreswamy S.M., Chakkarapani A.A., Murthy P. (2015). Saturation oxygen pressure index for assessment of pulmonary disease in neonates on non-invasive ventilation. Indian Pediatr..

[15] Bui-Binh-Bao S., Nguyen Thi D., Hoang Mai L., Do Ho Tinh T., Nguyen T.T.B. (2024). Assessing hypoxic respiratory failure in mechanically ventilated neonates: A comparative study of oxygen saturation index and oxygenation index. PLoS ONE.

[16] Tsurukawa S., Zuiki M., Naito Y., Kitamura K., Matsumura U., Kanayama T., Ichise E., Horiguchi G., Teramukai S., Komatsu H. (2024). Oxygenation saturation index in neonatal hypoxemic respiratory failure. Pediatr. Int..

[17] Khemani R.G., Rubin S., Belani S., Leung D., Erickson S., Smith L.S., Zimmerman J.J., Newth C.J.L. (2015). Pulse oximetry vs. PaO_2_ metrics in mechanically ventilated children: Berlin definition of ARDS and mortality risk. Intensive Care Med..

[18] Dargaville P.A., Aiyappan A., De Paoli A.G., Dalton R.G., Kuschel C.A., Kamlin C.O., Orsini F., Carlin J.B., Davis P.G. (2013). Continuous positive airway pressure failure in preterm infants: Incidence, predictors, and consequences. Neonatology.

[19] Krishnegowda S., Doreswamy S.M., Thandaveshwar D. (2017). Comprehensive, noninvasive saturation, oxygen, and pressure index: Does it reflect the severity of acute respiratory illness in neonates on continuous positive airway pressure? A prospective study. J. Clin. Neonatol..

[20] Horn-Oudshoorn E.J.J., Vermeulen M.J., Crossley K.J., Cochius-den Otter S.C.M., Schnater J.M., Reiss I.K.M., DeKoninck P.L.J. (2022). Oxygen Saturation Index in Neonates with a Congenital Diaphragmatic Hernia: A Retrospective Cohort Study. Neonatology.

[21] Htun Z.T., Schulz E.V., Desai R.K., Marasch J.L., McPherson C.C., Mastrandrea L.D., Jobe A.H., Ryan R.M. (2021). Postnatal steroid management in preterm infants with evolving bronchopulmonary dysplasia. J. Perinatol..

[22] Aziz K., Lee H.C., Escobedo M.B., Hoover A.V., Kamath-Rayne B.D., Kapadia V.S., Magid D.J., Niermeyer S., Schmölzer G.M., Szyld E. (2021). Part 5: Neonatal Resuscitation 2020 American Heart Association Guidelines for Cardiopulmonary Resuscitation and Emergency Cardiovascular Care. Pediatrics.

[23] Sweet D.G., Carnielli V.P., Greisen G., Hallman M., Klebermass-Schrehof K., Ozek E., Te Pas A., Plavka R., Roehr C.C., Saugstad O.D. (2023). European Consensus Guidelines on the Management of Respiratory Distress Syndrome: 2022 Update. Neonatology.

[24] Glenski J.A., Marsh H.M., Hall R.T. (1984). Calculation of mean airway pressure during mechanical ventilation in neonates. Crit. Care Med..

[25] Chou F.S., Leigh R.M., Rao S.S., Narang A., Yeh H.W. (2023). Oxygenation index in the first three weeks of life is a predictor of bronchopulmonary dysplasia grade in very preterm infants. BMC Pediatr..

[26] Jobe A.H., Bancalari E. (2001). Bronchopulmonary dysplasia. Am. J. Respir. Crit. Care Med..

[27] Jensen E.A. (2020). What is bronchopulmonary dysplasia and does caffeine prevent it?. Semin. Fetal Neonatal Med..

[28] Sunil B., Nithya E. (2021). Correlation of Oxygen Saturation Index and Oxygenation Index in Hypoxemic Respiratory Failure among Neonates. J. Clin. Diagn. Res..

[29] Durlak W., Thébaud B. (2024). BPD: Latest Strategies of Prevention and Treatment. Neonatology.

[30] Dini G., Ceccarelli S., Celi F. (2024). Strategies for the prevention of bronchopulmonary dysplasia. Front. Pediatr..

[31] Greenberg R.G., McDonald S.A., Laughon M.M., Tanaka D., Jensen E., Van Meurs K., Eichenwald E., Brumbaugh J.E., Duncan A., Walsh M. (2022). Eunice Kennedy Shriver National Institute of Child Health and Human Development Neonatal Research Network. Online clinical tool to estimate risk of bronchopulmonary dysplasia in extremely preterm infants. Arch. Dis. Child. Fetal Neonatal Ed..

[32] Kielt M.J., Logan J.W., Backes C.H., Conroy S., Reber K.M., Shepherd E.G., Nelin L.D. (2022). Noninvasive Respiratory Severity Indices Predict Adverse Outcomes in Bronchopulmonary Dysplasia. J. Pediatr..

[33] Xing W., He W., Li X., Chen J., Cao Y., Zhou W., Shen Q., Zhang X., Ta D. (2022). Early severity prediction of BPD for premature infants from chest X-ray images using deep learning: A study at the 28th day of oxygen inhalation. Comput. Methods Programs Biomed..

[34] Sharma A., Xin Y., Chen X., Sood B.G. (2020). Early prediction of moderate to severe bronchopulmonary dysplasia in extremely premature infants. Pediatr. Neonatol..

[35] Kostekci Y.E., Bakırarar B., Okulu E., Erdeve O., Atasay B., Arsan S. (2023). An Early Prediction Model for Estimating Bronchopulmonary Dysplasia in Preterm Infants. Neonatology.

[36] Srivatsa B., Srivatsa K.R., Clark R.H. (2023). Assessment of validity and utility of a bronchopulmonary dysplasia outcome estimator. Pediatr. Pulmonol..

[37] Jung Y.H., Jang J., Kim H.S., Shin S.H., Choi C.W., Kim E.K., Kim B.I. (2019). Respiratory severity score as a predictive factor for severe bronchopulmonary dysplasia or death in extremely preterm infants. BMC Pediatr..

[38] Di Fiore J.M., MacFarlane P.M., Martin R.J. (2019). Intermittent Hypoxemia in Preterm Infants. Clin. Perinatol..

[39] Jensen E.A., Whyte R.K., Schmidt B., Bassler D., Vain N.E., Roberts R.S. (2021). Canadian Oxygen Trial Investigators. Association between Intermittent Hypoxemia and Severe Bronchopulmonary Dysplasia in Preterm Infants. Am. J. Respir. Crit. Care Med..

